# "Safety First" Campaign

**Published:** 1920-10-02

**Authors:** 


### "Safety First" Campaign.

An important discussion took place in London a few days ago at a conference of employers and employed with regard to measures for preventing avoidable accidents in factories and workshops. The "safety first" propaganda campaign has been pursued with a good deal of energy, and the facts brought to light in this manner seem to indicate that Great Britain is not in the van of progress in the application of "safety first" knowledge and principles.

It may be perfectly true that it is practically impossible to eliminate from modern industry the variable human factor by which culpable negligence will sooner or later find a way to some accident that the most scientific safeguard cannot prevent. A fool-proof system is a phrase difficult to convert into an actuality in any industry. But the figures produced by Mr. Gerald Bellhouse (Deputy Chief Inspector of Factories) demonstrate that the number of accidents that take place annually in the factories and workshops of the kingdom are far above what they ought to be under any system of factory administration. During the year 1919 notices were received by inspectors attached to the Factory Department of the Home Office of 1,384 fatal accidents, 40,056 accidents due to machinery, and 84,582 non-machinery cases. Moreover, these are rather below the normal figures. A truer indication of the actual total is afforded by the return under the Workmen's Compensation Acts. In 1913, the last year for which complete returns have been compiled, there were in factories and docks 1,298 fatal accidents, for which £190,000 was paid in compensation, and 225,396 non-fatal cases, for which compensation amounting to £1,300,000 was paid.

Such figures as these prove the waste and loss of efficiency suffered every year by our industrial organisation through accidents alone. Yet it must be remembered, as Mr. Bellhouse himself admitted, that whatever may be accomplished by the provision of more adequate safeguards the reduction of accidents to be effected by this means alone could only represent a very small proportion of the whole. "In general accidents must be attributed to such causes as negligence, carelessness, want of instruction, want of thought, and perhaps more than all, want of appreciation of danger."

Official experience has shown that the number of accidents would be enormously reduced by the joint efforts of employers and workers in each individual trade and factory, and by the establishment of a definite safety organisation as a recognised part of the works management, and Mr. Bellhouse lays emphasis on the fact that the object of the "safety first" movement is to encourage the formation of organisations of this kind. Its function is mainly educational, and its aim is to reach individuals and to get all intelligently and sincerely interested in protecting themselves and their fellow-workers. It is claimed in America, we are told, as a result of twelve years' experience of safety work, that, given a proper safety organisation, 75 per cent. of all accidental deaths and serious injuries can be eliminated in industry. What America can do in this direction Great Britain can do; and if the worker is to be protected by ever-increasing legislation and fresh regulations against disease and undue fatigue, he must help the authorities to reduce this heavy casualty list in the ranks of our industrial army.

